# Tailored meta-analysis: an investigation of the correlation between the test positive rate and prevalence

**DOI:** 10.1016/j.jclinepi.2018.09.013

**Published:** 2019-02

**Authors:** Brian H. Willis, Dyuti Coomar, Mohammed Baragilly

**Affiliations:** a Institute of Applied Health Research, University of Birmingham, UK; b Department of Applied Statistics, Helwan University, Cairo, Egypt

**Keywords:** Data interpretation, Statistical, Decision making, Diagnosis tests, Routine, Mass screening, Meta-analysis, Models, Statistical

## Abstract

**Background and Objective:**

Meta-analysis may produce estimates that are unrepresentative of a test's performance in practice. Tailored meta-analysis (TMA) circumvents this by deriving an applicable region for the practice and selecting the studies compatible with the region. It requires the test positive rate, *r* and prevalence, *p* being estimated for the setting but previous studies have assumed their independence. The aim is to investigate the effects a correlation between *r* and *p* has on estimating the applicable region and how this affects TMA.

**Methods:**

Six methods for estimating 99% confidence intervals (CI) for *r* and *p* were investigated: Wilson's ± Bonferroni correction, Clopper-Pearson's ± Bonferroni correction, and Hotelling's T^2^ statistic ± continuity correction. These were analyzed in terms of the coverage probability using simulation trials over different correlations, sample sizes, and values for *r* and *p*. The methods were then applied to two published meta-analyses with associated practice data, and the effects on the applicable region, studies selected, and summary estimates were evaluated.

**Results:**

Hotelling's T^2^ statistic with a continuity correction had the highest median coverage (0.9971). This and the Clopper-Pearson method with a Bonferroni correction both had coverage consistently above 0.99. The coverage of Hotelling's CI's varied the least across different correlations. For both meta-analyses, the number of studies selected was largest when Hotelling's T^2^ statistic was used to derive the applicable region. In one instance, this increased the sensitivity by over 4% compared with TMA estimates using other methods.

**Conclusion:**

TMA returns estimates that are tailored to practice providing the applicable region is accurately defined. This is most likely when the CI for *r* and *p* are estimated using Hotelling's T^2^ statistic with a continuity correction. Potentially, the applicable region may be obtained using routine electronic health data.

**What is new?**

**Key findings**
• Meta-analysis may synthesize estimates that are unrepresentative of a test's performance in different settings.
• Routine data for the test positive rate and prevalence may help define an applicable region for a practice so that only those studies compatible with the region are selected for “tailored meta-analysis”.
• Previous research has ignored the effects of a potential correlation between the test positive rate and prevalence.

**What this adds to what was known?**
• Ignoring the correlation affects the size and shape of the applicable region and the number of studies selected for tailored meta-analysis, ultimately affecting the summary estimate.
• If Hotelling's T^2^ statistic is used to derive interval estimates for the test positive rate and prevalence then their correlation may be accommodated without knowing its exact level.

**What is the implication and what should change now?**
• When applying tailored meta-analysis in practice, we should not assume the test positive rate and prevalence, used to derive the applicable region for the practice, are independent.
• Importantly, their interval estimates should be based on Hotelling's T^2^ statistic to adequately accommodate their correlation.
• As more routine data are being collected as part of the electronic patient record, the potential to use tailored meta-analysis to inform diagnostic decisions in practice increases.

## 1 Introduction

It is of interest to policy makers and clinicians to ensure that the results of diagnostic tests from studies can be applied to a particular clinical setting. A diagnostic test's performance may be measured using several metrics—sensitivity, specificity, positive, or negative likelihood ratios [1], [2]. However, these metrics are influenced by many external factors such as disease prevalence, patient spectrum, test threshold, and reliability that sometimes change across different settings [3], [4], [5], [6], [7]. Traditional meta-analysis attempts to accommodate heterogeneity by pooling all the data in a random effects model [8], [9], [10], [11], [12], [13]. The bivariate random effects model is used to incorporate the sensitivity and specificity as the two outcomes of interest [8], [9], although others have used it to model the positive and negative predictive values [11]. Attempts to include additional information in the form of the prevalence have led to a trivariate model also being proposed [14], [15]. In general, these models only provide a single average estimate, and when there is heterogeneity it is unlikely to be representative of a particular clinical setting. Thus, it does not answer the specific question of whether the test estimate is representative of the performance of the test in a particular target setting.

Attempts have been made to address this problem, by tailoring the results of a meta-analysis to reflect the characteristics of the setting in question [16], [17], [18]. One solution is to estimate the test positive rate (*r*) and disease prevalence (*p*) from the setting where the results of the meta-analysis are to be applied [16], [17]. Then, using interval estimates of *r* and *p*, a region of feasible values for the sensitivity (*s*) and the false positive rate (*f*) can be deduced for the test in receiver operating characteristic (ROC) space. Studies are only included in the meta-analysis if their sensitivities and FPRs lie close to or within the plausible or “applicable region” for the test in the setting.

Empirically, the test positive rate may be estimated from data on the number of patients testing positive in those who are tested. National screening programmes already do this routinely [19], [20], and with greater uptake of electronic records being used to code patient data in primary and secondary care, this is becomingly increasing possible in these settings. The prevalence is likely to be more difficult to estimate and may be obtained from verifying a subsample of patients (if the reference standard is available) or in some cases from local laboratory data. Within a Bayesian framework, both of these statistics may be based on degrees of belief.

To accurately ascertain the applicable region, the interval estimates for the test positive rate and prevalence need to contain the true parameters. Although in general, this can only be achieved with the interval [0,1], interval estimates with a high coverage probability may be used. Furthermore, the narrower the intervals, the smaller the applicable region, and the more informative it is on where the test performance in ROC space lies for the setting in question.

Previous analyses have treated the test positive rate and prevalence as independent when calculating the confidence intervals [16], [17]. To ensure a high coverage, 99% confidence intervals are usually chosen so that if an interval has been estimated for each of *r* and p then the joint coverage probability will be 0.99^2^, that is, 0.9801. However, in practice the test positive rate and prevalence are likely to be correlated potentially reducing the joint coverage probability of interval estimates obtained independently.

To preserve a high joint coverage probability, it is likely that any potential correlation between *r* and *p* needs to be considered when estimating the confidence intervals. Moreover, the resulting confidence intervals will modify the size and shape of the applicable region and studies selected for tailored meta-analysis as a result.

Thus, the aim of this study is to investigate the effects the correlation between the test positive rate and prevalence has on estimating the applicable region and how this affects tailored meta-analysis.

## 2 Methodology

### 2.1 Defining an applicable region for the setting

Tailored meta-analysis relies on using routine data from the setting of interest to define an applicable region for the test to select the relevant studies. In particular, if we have 99% confidence interval estimates for the test positive rate parameter, $\mu_{r}$, and the prevalence parameter, $\mu_{p}$, for the setting such that $r_{lcl}\leq \mu_{r}\leq r_{ucl}\;\text{and}\;p_{lcl}\leq \mu_{p}\leq p_{ucl}$ (where *lcl* an *ucl* refer to the lower and upper confidence limits) then for $\mu_{s}\;\text{and}\;\mu_{f}$, the parameters for the sensitivity and false positive rate, respectively, the following inequalities allow us to derive an applicable region in ROC space:

(1) $$0\leq \mu_{f}\leq \mu_{r}\leq \mu_{s}\leq 1$$

(2) $$\mu_{s}\leq \frac{r_{ucl}}{p_{lcl}}-\frac{\left(1-p_{lcl}\right)\mu_{f}}{p_{lcl}}$$

(3) $$\mu_{s}\geq \frac{r_{lcl}}{p_{ucl}}-\frac{\left(1-p_{ucl}\right)\mu_{f}}{p_{ucl}}$$

Thus, these inequalities (1)–(3) constrain the feasible values for $\mu_{f}\;\text{and}\;\mu_{s}$ to a subspace of the [0,1] × [0,1] plane that is ROC space. This is the applicable region for the test in the setting. The derivation and justification of the inequalities (1)–(3) may be found in Appendix 1 in [16] and Appendix 1 in [17].

The truth of (2) and (3) depends on $r_{lcl}\leq \mu_{r}\leq r_{ucl}\;\text{and}\;p_{lcl}\leq \mu_{p}\leq p_{ucl}$ being true. In practical terms, this means choosing confidence intervals for $\left(\mu_{r},\;\mu_{p}\right)$ that have a high coverage probability, so that in the long run a high proportion of the intervals contain the parameters $\left(\mu_{r},\mu_{p}\right)$. As a minimum requirement, 99% confidence intervals have been suggested, although the higher the coverage probability the better, and this is likely to be affected by any correlation between $\mu_{r}\;\text{and}\;\mu_{p}$. Here, the coverage probabilities of 99% confidence intervals derived using the different methods below will be investigated.

### 2.2 Joint distribution

Let X be the number who test positive in a sample of $n_{r}$ individuals and Y be the number with the target disorder in a sample of $n_{p}$ individuals. For marginal distributions that are binomial, the joint distribution of X and Y is given by

(4) $$\left(X,Y\right)∼\;Bivariate\;Binomial\left(\mu_{r},\;n_{r},\;\mu_{p},n_{p},\rho \right)$$

where $\mu_{r}\;\text{and}\;\mu_{p}$ are the parameters for the test positive rate and prevalence, respectively, with correlation ρ.

### 2.3 Assuming independence

When the proportions of interest are independent, univariate confidence intervals may be estimated. Here, two methods were used. The first, the Wilson's score method [21] has been used in previous studies [16], [17], and the second, the Clopper-Pearson interval is sometimes known as an “exact” interval [22]. Both are briefly described below.

#### 2.3.1 Wilson's score method

Strictly the variance for the asymptotic normal distribution for a proportion is *μ(1-μ)/n* where *μ* is the true proportion parameter for the population. Although this is unknown, Wilson's method [21] overcomes this by solving for *μ* explicitly in terms of the sample estimate $\hat{p}$, the sample size *n,* and the *z* score for level of significance *α*. This allows us to estimate a confidence interval for a proportion, and this was used to provide interval estimates for the test positive rate and prevalence (see Appendix).

For a single 99% confidence interval, Wilson's score method is efficient and is known to have a coverage probability close to 0.99 [23]. However, for two simultaneous confidence intervals, this does mean the coverage probability is likely to be below 0.99 and vary with the correlation.

#### 2.3.2 Clopper-Pearson interval

For a sample size *n*, with *k* successes a 100 (1-α)% confidence interval $\left[\mu_{L},\mu_{U}\right]$ may be found by solving the two equations $P\left(X\geq k\text{|}\;\mu_{L},n\right)=\;\alpha /2$ and $P\left(X\leq k\text{|}\mu_{U},n\right)=\alpha /2$ for $\mu_{L}$ and $\mu_{U}$. When X has a binomial distribution, this provides us with the Clopper-Pearson interval [22]. However, because the binomial distribution is discrete, it is not always possible to find $\mu_{L}$ and $\mu_{U}$ that satisfy these equations. Hence, a related continuous distribution, the beta distribution, may be used to estimate the Clopper-Pearson intervals for the test positive rate and prevalence (see Appendix).

The Clopper-Pearson confidence intervals are known to be conservative producing coverage probabilities greater than 0.99 [23]. This latter property will benefit simultaneous intervals where the coverage although lower than 0.99 is higher than the coverage from simultaneous confidence intervals using Wilson's score method.

### 2.4 Including correlation between proportions

If the test positive rate and prevalence are treated as independent when they are correlated then the resulting interval estimates may have inadequate coverage probabilities. Two methods for estimating simultaneous confidence intervals in correlated variables were used and are described below.

#### 2.4.1 Confidence intervals using Hotelling's $T^{2}$ statistic

Hotelling's T^2^ distribution is a multivariate generalization of Student's t distribution allowing the study of correlated variables [24]. Using this distribution, a rectangle with dimensions equal to the width of the confidence intervals may be derived that neatly contains the elliptical cross-section of a bivariate distribution. As it is continuous and unbounded, the logit transformation of the test positive rate, *r* and prevalence, *p* was used. The variance was estimated using the delta method [25]. The formulae for the confidence intervals are given in the Appendix.

Cell entries within the 2 by 2 contingency table that were a zero were accommodated by adding 0.5 as an ad hoc continuity correction.

#### 2.4.2 Bonferroni procedure

The Bonferroni procedure adjusts the level of significance, *α* for each interval estimate to ensure adequate joint coverage probability [26]. Since for 2 events A and B, $P\left(A\cap B\right)\geq 1-\left(P\left(A^{c}\right)+P\left(B^{c}\right)\right)$, then we may set $P\left(A^{c}\right)=P\left(B^{c}\right)=\alpha$ so that P(A∩B)≥1−2α. Thus, if A and B are the events that the interval estimates cover their respective parameters, then setting α=0.005 will provide a joint coverage probability of at least 99% for A and B. This method was used to modify the Wilson's score interval and the Clopper-Pearson interval estimates for both the test positive rate and prevalence.

### 2.5 Simulation study

A simulation study was conducted to evaluate the coverage probabilities of the different methods (Wilson's score with and without a Bonferroni correction, Clopper-Pearson with and without a Bonferroni correction, and Hotelling's T^2^ with and without a continuity correction) for estimating 99% confidence intervals for the test positive rate and prevalence. A random sample from the bivariate binomial distribution in (4) was generated using a copula [27]. This is a generalization of the inverse probability integral transformation, which allows any distribution to be simulated from first simulating from a uniform distribution. A copula extends this idea to multivariate distributions by treating each marginal distribution of the joint cumulative distribution as uniform and capturing the correlation between the variables.

Simulated observations of the variables (X,Y) were generated for different values of the following parameters: $\mu_{r}$ (the test positive rate parameter), $n_{r}$ (the sample size in which the test positive rate is calculated), $\mu_{p}$ (the prevalence parameter), $n_{p}$ (the sample size in which the prevalence is calculated), ρ (the correlation coefficient between $\mu_{r}$ and $\mu_{p}$). For each of these the following values were used $\mu_{r}$[0.1, 0.25, 0.5, 0.75, 0.9]; $n_{r}$[25, 50, 100, 500, 1000]; $\mu_{p}$[0.1,0.25,0.5,0.75,0.9]; $n_{p}$[25,50,100,500,1000]; ρ[0, 0.1,0.25,0.5,0.75,0.9,1]. For different combinations of $\left(\mu_{r},\;n_{r},\;\mu_{p},\;n_{p},\;\rho \right)$, the coverage probability for the confidence intervals calculated using each of the four different methods was estimated for a critical value of 0.01. The estimates of the coverage probabilities were based on 100,000 replications for each $\left(\mu_{r},\;n_{r},\;\mu_{p},\;n_{p},\;\rho \right)$ combination.

### 2.6 Case studies

To illustrate the effects the different methods have on tailored meta-analysis, two data sets from a previous published study were used [17]. The first was a meta-analysis used to assess the accuracy of the PHQ-9 to screen for depression in primary care. Data collected from a UK general practice were used to calculate an interval estimate of the test positive rate for that practice. For the prevalence of depression in the practice population of interest, the previous interval estimate [17] was updated by using practice-specific routine data, which are collected as part of quality of outcomes framework (QOF). The QOF data form part of the electronic record in general practice surgeries in the UK and are available in the public domain for each practice [28]. A 99% confidence interval was estimated based on 350 patients with a diagnosis of depression from 5,365 eligible patients in the practice [28].

In the second case, the meta-analysis investigated the accuracy of Centor's criteria in diagnosing streptococcal infection in those presenting to primary care with a sore throat. Previously collected data from the same UK general practice were used to estimate the test positive rate and prevalence for the practice [17].

### 2.7 Statistical analyses

All summary sensitivity and specificity estimates were derived using the bivariate random effects model [9]. All analyses were conducted in R [29].

## 3 Results

### 3.1 Simulation study

**Fig. 1:**
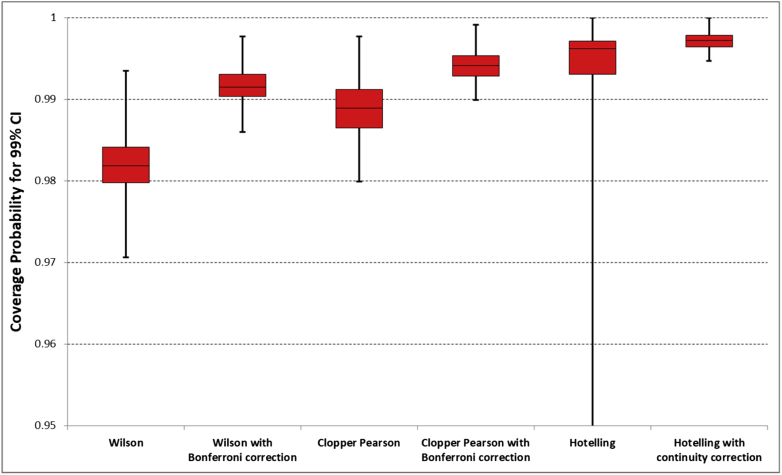
Distribution of coverage probabilities for the 99% confidence interval over the different combinations of $\left(\mu_{r},\;n_{r},\;\mu_{p},\;n_{p},\;\rho \right)$. For each method the box and whisker comprises the minimum, lower quartile, median, upper quartile, and maximum.

In Figure 1, the distribution of coverage probabilities over all the combinations of $\left(\mu_{r},\;n_{r},\;\mu_{p},\;n_{p},\;\rho \right)$ is given for each of the different methods in estimating a 99% confidence interval. From the figure, it is clear that Wilson's score method rarely (0.3%) achieves the required coverage probability of 0.99; the coverage probability of Wilson's score method is improved with a Bonferroni correction, with a median of 0.9926 but it is still less than 0.99 in over 20% of cases, with a minimum of 0.986. Without the continuity correction, 99% confidence intervals estimated using Hotelling's T^2^ statistic may have a coverage probability as low as 0.86. This tends to occur when the prevalence or test positive rate is 0.1. With the continuity correction, Hotelling's T^2^ statistic produces confidence intervals with high coverage probabilities, median = 0.9972, minimum 0.9947. The Clopper-Pearson interval with a Bonferroni correction has coverage probability above 0.99 in all but 0.02% of cases with a median of 0.9941.

Table 1 provides a breakdown of the coverage probabilities per method according to the test positive rate parameter and the sample size. While these two quantities do affect the coverage probability of confidence intervals derived using Hotelling's method, the coverage probabilities of the other methods are largely unaffected by them.

**Table 1:** Mean coverage probability of each method according to the test positive rate and sample size

*μ*_*r*_	Wilson score		Clopper-Pearson		Hotelling T^2^	
	Standard	Bonferroni	Standard	Bonferroni	Standard	Continuity
0.1	0.98154	0.99175	0.98872	0.99423	0.97585	0.99697
0.25	0.98189	0.99120	0.98824	0.99383	0.99083	0.99712
0.5	0.98091	0.99070	0.98793	0.99330	0.99108	0.99725
0.75	0.98191	0.99122	0.98824	0.99383	0.99085	0.99712
0.9	0.98154	0.99178	0.98870	0.99422	0.97581	0.99697
*n*						
25	0.98018	0.99247	0.99094	0.99467	0.96374	0.99825
50	0.98291	0.99147	0.98996	0.99487	0.98949	0.99758
100	0.98140	0.99013	0.98867	0.99343	0.99070	0.99686
500	0.98142	0.99123	0.98580	0.99335	0.99030	0.99643
1000	0.98188	0.99135	0.98646	0.99307	0.99019	0.99632

**Fig. 2:**
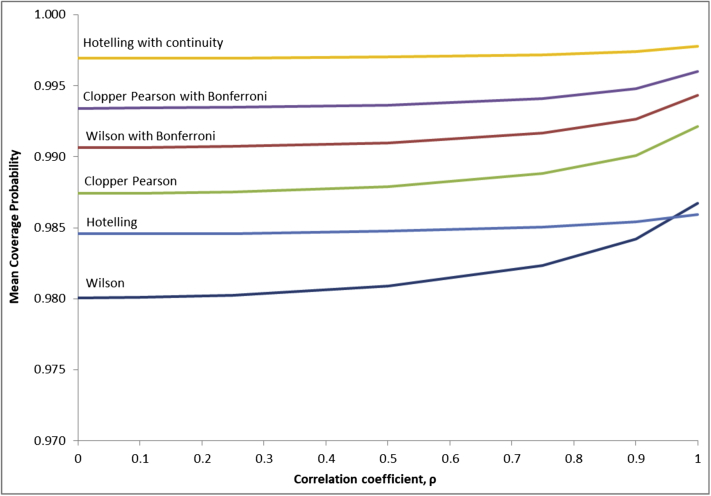
Mean coverage probability as a function of correlation.

Figure 2 illustrates the effects of a correlation between the test positive rate and prevalence has on the mean coverage probabilities. Using Hotelling's method both with and without a continuity correction, the coverage probability remains relatively constant as the correlation changes. For the other methods, there is a small rise ranging between 0.25% and 0.67% as the correlation increases from 0 to 1.

### 3.2 Case studies

Tables 2 and 3 demonstrate the effect the methods have on the selection of studies for the purpose of tailored meta-analysis. There were 12 and 10 studies meeting the qualitative inclusion criteria for each of the cases. The narrower the interval estimates for $\mu_{r}$ and $\mu_{p}$, the narrower the applicable region for the setting of interest, and the lower the probability of study inclusion for tailored meta-analysis.

**Table 2:** Tailored selection for meta-analysis of Centor’s criteria according to method used

Study	All	Wilson	Wilson Bonferroni	Clopper Pearson	Clopper Pearson Bonferroni	Hotelling	Hotelling Continuity
Fine	Include				Include	Include	Include
Regueras	Include						
Canada	Include						
Treebupachatsak	Include	Include	Include	Include	Include	Include	Include
Atlas	Include					Include	Include
Dagnelie	Include						
Hall et al	Include	Include	Include	Include	Include	Include	Include
Scwartz et al	Include	Include	Include	Include	Include	Include	Include
Seppala et al	Include	Include	Include	Include	Include	Include	Include
McIsaac	Include						
Alper et al	Include	Include	Include	Include	Include	Include	Include
Abu-Sabaah et al	Include						
Sensitivity (95% CI)	50.6% (42.8-58.4)	38.4% (30.4-47.2)	38.4% (30.4-47.2)	38.4% (30.4-47.2)	39.7% (31.5-48.6)	42.4% (35.2-49.9)	42.4% (35.2-49.9)
Specificity (95% CI)	78.5% (65.7-87.4)	92.1% (83.5-96.4)	92.1% (83.5-96.4)	92.1% (83.5-96.4)	89.5% (82.4-94.0)	88.4% (79.4-93.8)	88.4% (79.4-93.8)

**Table 3:** Tailored selection for meta-analysis of the PHQ-9 tool according to method used

Study	All	Wilson	Wilson Bonferroni	Clopper-Pearson	Clopper- Pearson Bonferroni	Hotelling	Hotelling Continuity
Arroll	Include	Include	Include	Include	Include	Include	Include
Ayalon	Include						
Azah	Include	Include	Include	Include	Include	Include	Include
Cheng	Include	Include	Include	Include	Include	Include	Include
Zuithoff	Include						
Gilbody	Include	Include	Include	Include	Include	Include	Include
Lotrakul	Include	Include	Include	Include	Include	Include	Include
Inagaki	Include						
Liu	Include				Include	Include	Include
Sherina	Include	Include	Include	Include	Include	Include	Include
Sensitivity (95% CI)	74.2% (63.2-82.8)	78.8% (69.7-85.7)	78.8% (69.7-85.7)	78.8% (69.7-85.7)	79.7% (71.6-86.0)	79.7% (71.6-86.0)	79.7% (71.6-86.0)
Specificity (95% CI)	91.5% (86.5-94.8)	86.3% (81.4-90.1)	86.3% (81.4-90.1)	86.3% (81.4-90.1)	87.9% (83.1-91.5)	87.9% (83.1-91.5)	87.9% (83.1-91.5)

In each case, the methods that resulted in the fewest studies being included for tailored meta-analysis were those that had the three lowest median coverage probabilities: Wilson's score method; Wilson's score method with a Bonferroni correction; and the Clopper-Pearson method. When implementing each of these three methods, study selection was tailored to 5/12 and 6/10 studies for the two cases, respectively. Adding a Bonferroni correction to the Clopper-Pearson method resulted in a further study being included in each case, while there were seven studies included in each case when the confidence intervals were derived using Hotelling's T^2^ statistic with and without a continuity correction.

The effects of the different methods on the summary estimates are also given. As previously reported, tailoring the study selection can have a substantial effect on the summary sensitivity and specificity compared with a conventional estimate. Second, it is clear that the method used to derive the intervals may have a modest effect on the summary estimates; the largest difference was a 4% difference in the sensitivity between using Wilson's score and Hotelling's T^2^ statistic for Centor's criteria.

In the two case examples, Pearson's correlation coefficient was estimated to be 0.75 and 0.94, respectively. For the non-Hotelling methods, these correspond to correlations where the mean coverage probability changes with the correlation coefficient (figure 2); thus any uncertainty in the latter will introduce uncertainty in the coverage probability.

## 4 Discussion

Meta-analysis is used to aggregate test accuracy studies to produce a quantitative summary estimate of the test's performance. Although this may be useful in some circumstances, it is important to remember that it represents an average across all studies and may not be representative of an individual study. This is pertinent to the problem of determining when to apply the results to clinical practice. The test, when applied in a particular practice setting, may have a sensitivity and specificity which is in a different region of ROC space to that reported for the summary estimate. This clearly has implications for clinical decision-making.

To overcome this, tailored meta-analysis has been proposed. This uses information from the setting of interest to define an applicable region for the test and combines this with the studies from the meta-analysis so that only those studies that are compatible with the region are selected. This enables a summary estimate to be derived that is tailored to the setting.

Thus, it is important that the applicable region accurately defines the region in ROC space for the test in the setting of interest and this depends on the accuracy of our estimates for the test positive rate and the prevalence. In practice, this means the interval estimates require a high coverage probability, and a minimum of a 99% confidence interval has been suggested. However, what has not been considered until now is the effect the potential correlation between the prevalence of disease and the test positive rate for the test may have on the methods reported in previous studies.

In previous analyses, when these two parameters have been treated as independent, Wilson's score method has been used to derive a 99% confidence interval. Without modification this method returns the lowest joint coverage probability in the simulation analyses. The effect of this is that the parameters $\mu_{r}$ and ${\text{μ}}_{p}$ may lie outside of their respective interval estimates and the probability of the inequalities (2) and (3) not being satisfied increases. In effect, the applicable region is narrower than is necessary to adequately represent the test performance in the setting of interest. Although the Clopper-Pearson's “exact” interval has better coverage than Wilson's score method, without a Bonferroni correction it too has coverage less than 99% in the majority of cases (nearly 70%).

Here, we used two methods to improve the coverage probability. The first, the Bonferroni correction modifies the levels of significance for the individual intervals to produce a joint coverage probability at the desired level. This improved both the Wilson's score method and the Clopper-Pearson's method. The second, Hotelling's T^2^ statistic is a multivariate generalization of student's t statistic. Without a continuity correction, when one or more of the cell entries in the 2 × 2 table contain a zero, its coverage may be erratic. However, with a continuity correction, the coverage is more conservative and always above 99%.

When applied to the two clinical cases, it is clear that the method used to estimate $\mu_{r}$ and $\mu_{p}$ may change the shape of the applicable region sufficiently to affect the number of studies included in the tailored meta-analysis. Ultimately this may affect the tailored estimate. For example, the tailored estimates for the sensitivity and specificity of Centor's criteria change by over 3% between methods.

Furthermore, the simulation analyses reveal that the coverage probability remains relatively constant across different values of the correlation when using the Hotelling statistic and increases slightly with correlation for the other methods. In practical terms, this means that not knowing the true correlation between the test positive rate and disease prevalence in the setting of interest does not pose a significant problem.

So which method should we use? Because the mathematical truth of the inequalities in (2) and (3) relies on the parameters $\mu_{r}$ and $\mu_{p}$ being covered by their respective interval estimates, it is imperative that whichever method is used, the risk of violating either inequality is kept to a minimum. Essentially there are only two methods where this risk is consistently below 1%: the Clopper-Pearson with a Bonferroni correction, where the risk is above 1% in only 0.02% of cases and Hotelling's T^2^ statistic with a continuity correction, where the risk is always below 1%. The decision on which method to use rests on weighing up the need for maintaining the highest possible coverage probability and therefore the lowest risk of violating (2) or (3) and a more informative (narrower) applicable region for selecting studies.

On balance, we recommend using the Hotelling's T^2^ statistic with a continuity correction for estimating the 99% confidence intervals for the test positive rate and prevalence. This is because it provides the lowest risk (maximum = 0.53%) of violating (2) or (3), has a coverage probability that varies the least with correlation, and it helps define an applicable region that includes only one more study in one of the tailored meta-analysis examples than the next best method.

Although tailored meta-analysis provides a summary estimate for the test which is more specific to the clinical setting, it is worth stating that this is still just a feasible estimate given the combined information from the included studies and the test positive rate and prevalence of disease for the setting. Feasibility does not necessarily translate into accuracy as the included studies, although feasible for the setting, may not be representative. This is because of part of the study selection process being probabilistic and so estimates which may appear compatible with the applicable region may do so due to random error. Thus, it is important to consider not just the effects the applicable region has on the summary estimate but also on its associated confidence region.

To improve the accuracy of estimates for a particular setting requires methods which assess their validity and this is a source of active research. Validation statistics, such as the *Vn* statistic, have been proposed recently as a means of checking the validity of estimates from univariate meta-analyses [30]. Other methods involve estimating prediction regions in an attempt to quantify the error in the predicted estimates from meta-analyses [18]. Both approaches need further development.

Previous studies [16], [17] have shown that for some tests the data required to derive an applicable region are already being collected routinely. This is the case with the UK national screening programmes for cervical cancer and breast cancer [19], [20]. As the use of electronic health records increases, there is an opportunity for this to extend to other tests in primary and secondary care. In the original study, the applicable regions for Centor's criteria and the PHQ-9 were derived using ad hoc data collected for the purpose of tailored meta-analysis [17]. In this study, the prevalence of depression was estimated from the routine data collected as part of QOF. Clinical templates that allow the data necessary for a questionnaire or prediction rule to be input directly to the electronic record are already available [31]. This opens a possible future in which applicable regions for a practice are derived completely from routine electronic health data. In such an instance, tailored meta-analysis will truly represent the combining of routine data with published research to inform clinical decisions.

In summary, tailored meta-analysis provides a means of deriving summary estimates for the sensitivity and specificity of a test, which are tailored to clinical practice. It involves defining an accurate applicable region in ROC space, using routine data to calculate 99% confidence intervals for the test positive rate, *r* and disease prevalence, *p* in the setting. The use of Wilson's score method to calculate these intervals, as used in previous studies [16], [17], is not recommended because of a potential correlation between *r* and *p*. Instead, Hotelling's T^2^ statistic with a continuity correction should be used as this is most likely to lead to an applicable region, which accurately represents the setting while still being useful to decisions on study selection for tailored meta-analysis.

## Appendix: Confidence intervals for binomial proportions

### (i) Wilson's confidence intervals

If μ is the population parameter for the mean proportion, the variance for the asymptotic normal distribution is *μ(1-μ)/n.* For a sample estimate of the test positive rate, *r*, of sample size $n_{r}$, and standardised normal variable z, at the upper and lower bounds,

$$\mu =r\;\pm z\sqrt{\frac{\mu \left(1-\mu \right)}{n_{r}}}$$

Solving for μin terms of r, z, and $n_{r}$ gives the following 99% confidence interval for the test positive rate, *r* with *z* = 2.576

$$\left(\frac{r+\;\frac{z^{2}}{2n_{r}}-\;z\sqrt{\frac{r\left(1-r\right)}{n_{r}}+\frac{z^{2}}{4n_{r}^{2}}}}{\left(1+\frac{z^{2}}{n_{r}}\right)},\;\;\frac{r+\;\frac{z^{2}}{2n_{r}}+\;z\sqrt{\frac{r\left(1-r\right)}{n_{r}}+\frac{z^{2}}{4n_{r}^{2}}}}{\left(1+\frac{z^{2}}{n_{r}}\right)}\right)$$

Similarly a confidence interval for the prevalence may be derived.

### (ii) Clopper-Pearson's confidence intervals

To estimate the Clopper-Pearson confidence interval [18] a connection between the discrete binomial distribution and the continuous beta distribution is exploited. Essentially for a sample of size, *n* drawn from a *Uniform[0, 1],* the number of draws, *k*, with a value less than or equal to probability *p*, has a *Binomial(n, p)* distribution. Moreover, if the sample of draws from the uniform distribution is ordered in terms of magnitude, the resulting ordered statistic has a Beta(α,k,n−k+1) distribution for level of significance. Because in general, P(X≤k)=1−P(X>k) and for a binomial where the *k* values are discrete P(X≤k)=1−P(X≥k+1), the confidence interval at level of significance, α is given by

[Beta(α/2,k,n−k+1),Beta(1−α/2,k+1,n−k)]

Thus the 99% confidence interval for the test positive rate *r = k/*$n_{r}$*,* for sample size, $n_{r}$ is given by

$$\left[Beta\left(0.005,k,n_{r}-k+1\right),\;\;Beta\left(0.995,k+1,n_{r}-k\right)\right]$$

Similarly, the 99% confidence interval for the prevalence may be derived.

### (iii) Confidence intervals using Hotelling's T^2^ distribution

Let x be the vector of observed values for 2 correlated variables, μ be the associated parameter vector and *S* be the sample covariance matrix. The Hotelling statistic $T^{2}$ is defined as $T^{2}=\left(n-1\right){\left(x-\mu \right)}^{T}S^{-1}\left(x-\mu \right)$ [19] and has the following distribution:

$$T^{2}∼\frac{2\left(n-1\right)}{\left(n-2\right)}F_{2,\;n-2}$$

Thus, it is proportional to the F distribution. The confidence interval for the *i*th element of x at a level of significance *α* is given by

$$\left(x_{i}-\sqrt{\frac{2\left(n-1\right)}{\left(n-2\right)}F_{2,\;n-2,\left(1-\text{α}\right)}S_{ii}\;},\;\;x_{i}+\sqrt{\frac{2\left(n-1\right)}{\left(n-2\right)}F_{2,\;n-2,\left(1-\text{α}\right)\;}S_{ii}\;}\right)$$

Here $x_{1}$ is logit(r)and $x_{2}$ is logit(p). For sample size $n_{r}$, $S_{11}$ is the associated variance for the logit(r) estimated by the delta method to be $1/n_{r}\left(1-r\right)r$ [20]. Similarly, $S_{22}=$$1/n_{p}\left(1-p\right)p$ for sample size $n_{p}$.

For the logit(r), the 99% confidence interval is given by

$$\left(logit\left(r\right)-\sqrt{\frac{2\left(n_{r}-1\right)F_{2,\;n_{r}-2,0.99}}{\left(n_{r}-2\right)n_{r}\left(1-r\right)r}},\;\;logit\left(r\right)+\sqrt{\frac{2\left(n_{r}-1\right)F_{2,\;n_{r}-2,0.99\;}}{\left(n_{r}-2\right)n_{r}\left(1-r\right)r}}\right)$$
